# Evaluation of Mechanical Properties of ABS-like Resin for Stereolithography Versus ABS for Fused Deposition Modeling in Three-Dimensional Printing Applications for Odontology

**DOI:** 10.3390/polym16202921

**Published:** 2024-10-17

**Authors:** Victor Paes Dias Gonçalves, Carlos Maurício Fontes Vieira, Noan Tonini Simonassi, Felipe Perissé Duarte Lopes, George Youssef, Henry A. Colorado

**Affiliations:** 1 Advanced Materials Laboratory—LAMAV, State University of the Northern Rio de Janeiro—UENF, Av. Alberto Lamego, 2000, Campos dos Goytacazes 28013-602, RJ, Brazil; 2 Experimental Mechanics Laboratory, Department of Mechanical Engineering, San Diego State University, 5500 Campanile Dr., San Diego, CA 92182, USA; 3 CCComposites Laboratory, Engineering School, Universidad de Antioquia (UdeA), Calle 70 No. 52-21, Medellin, CO 050010, USA

**Keywords:** acrylonitrile butadiene styrene, polymer, 3D printing, odontology

## Abstract

This study investigates the differences in mechanical properties between acrylonitrile butadiene styrene (ABS) samples produced using fused deposition modeling (FDM) and stereolithography (SLA) using ABS filaments and ABS-like resin, respectively. The central question is to determine how these distinct printing techniques affect the properties of ABS and ABS-like resin and which method delivers superior performance for specific applications, particularly in dental treatments. The evaluation methods used in this study included Shore D hardness, accelerated aging, tensile testing, Izod impact testing, flexural resistance measured by a 3-point bending test, and compression testing. Poisson’s ratio was also assessed, along with microstructure characterization, density measurement, confocal microscopy, dilatometry, wettability, Fourier-transform infrared spectroscopy (FTIR), and nanoindentation. It was concluded that ABS has the same hardness in both manufacturing methods; however, the FDM process results in significantly superior mechanical properties compared to SLA. Microscopy demonstrates a more accurate sample geometry when fabricated with SLA. It is also concluded that printable ABS is suitable for applications in dentistry to fabricate models and surgical guides using the SLA and FDM methods, as well as facial protectors for sports using the FDM method.

## 1. Introduction

Acrylonitrile butadiene styrene (ABS) is the most common thermoplastic material in multiple applications and is ubiquitous in three-dimensional printing (3DP) technology. ABS is a terpolymer comprising three monomers: acrylonitrile, butadiene, and styrene [1]. ABS is quite popular in many applications because it supports higher temperatures than other thermoplastics and has good mechanical strength, which makes it the most affordable thermoplastic in the 3D printing filament market. It also has good electrical insulation, chemical resistance properties, and durability [1]. For 3DP, ABS has excellent adhesion between the printed layers, pleasant aesthetics, minimal deformation, and high durability [1]. However, the risks that printing can cause are also currently being investigated. A study related to the emission of ultrafine aerosol [2] shows that ABS is the most toxic, with the styrene component responsible for toxins emission, i.e., the emission of volatile organic compounds (VOC) [3,4].

Three-dimensional printing (3DP), or Additive Manufacturing (AM), is a modern fabrication technology that has evolved rapidly over its nearly forty-year history. The AM approach differs from classical subtractive manufacturing principles by adding materials layer-by-layer instead of removing materials from the bulk workpiece, and it is currently used in many practical applications. The classification of AM technologies is presented in the literature and divided into seven groups: material extrusion; powder bed fusion; binder jetting (a liquid binder is deposited in thin layers of material powder); sheet lamination; material jetting (deposition of fluid material, layer-by-layer, using print heads like those of an inkjet printer); vat photopolymerization; and direct energy deposition [5]. Additive manufacturing is a very versatile technology that can be applied to all types of materials, such as cement [6], thermosetting resins [7], metals [8], and thermoplastics [9], among many others. The applications also vary broadly from education [10] and dentistry [11] to transportation [12].

The AM fabrication scheme allows for rapidly creating complex custom parts [13,14]. Wong and Pfahnl [1] used ABS to print surgical instruments such as sponges, towel clamps, scalpel handles, and toothed forceps. Surgeons agreed that printed smooth and textured forceps would perform adequately in simulated surgical tasks. Li et al. [15] studied the effects of sterilizations on the mechanical behavior of 3D-printed ABS parts since such conditions are commonly applied to medical instruments in a hospital environment, which may alter the instrument’s functionality [16]. However, the results indicated no significant differences between the strength and stiffness of sterilized and non-sterilized specimens [16].

The possibility of 3D printing in dental treatments progressively demands innovation and quality since companies seek to invest in new materials and processes, including digital manufacturing technologies, to accelerate advancements into Industry 4.0 [17]. AM technologies increasingly facilitate, speed up, and improve the digital workflow, allowing optimization, high quality, and high productivity. A few areas of 3D printing have evolved to impact progress in the industry in recent years, such as dentistry, which is moving along with globalization and offering different resources and solutions [18]. Hence, 3D printing is becoming exceedingly popular among medical professionals and practitioners. McGlumphy et al. [18] and Liang et al. [19], for example, agree that 3DP can reduce material waste and costs while improving the accuracy of the fabricated parts and making this paradigm attractive.

Among the 3DP technologies, material extrusion is a relatively cheap and popular manufacturing technique that can be used to fabricate complex 3D geometries [20]. Fused filament manufacturing (FFF), or Fused deposition modeling (FDM), is the most widespread 3DP technology, especially among non-industrial users [21]. FFF is also accepted as an attractive alternative to conventional manufacturing because of several intrinsic and well-documented advantages: convenience, versatility, multi-material parts, and shortened production time [20]. However, parts produced by this process are plagued by low dimensional and geometric accuracy, primarily due to thermal cycling during printing. Moreover, Z-axis resolution, surface finish (printing speed and extrusion rate), and controlling the printing environment temperature also significantly inhibit the uptake of production quality components related to achieving dimensional quality (DQ).

Stereolithography (SLA), also known as vat photopolymerization (VPP), is another typical 3DP process where photocurable resins are cured using ultraviolet LED or laser sources to initiate radical polymerization via photo-initiators, resulting in conversion into a solid polymer [22,23,24,25]. SLA exists in different variations based on the light source type and printing configuration. For example, digital light processing (DLP) technology uses a digital projector to send an image (a slice) of the object layer to induce photopolymerization of the resin. Thus, DLP projects a mask of the layer to be printed at once while obscuring exposure of the remaining resin to maintain its integrity for subsequent printing steps. The exposed resin hardens via photopolymerization, while the viscosity of the uncured resin might evolve due to the light-scattering phenomenon [21]. SLA and its variations, produce parts at faster speeds than FFF. As discussed above, the SLA-printing method uses a photopolymer resin that induces free-radical photopolymerization in acrylates [26], where the photo-initiator compounds cleave the side groups to initiate and propagate the polymerization process and build the molecule by adding segments of monomers [26,27]. These molecules often exhibit poor mechanical properties due to the nature of the substance and the polymerization process. SLA-printed polymers are also susceptible to ultraviolet radiation, temperature, and humidity, to name a few detrimental factors [28,29]. Nonetheless, the SLA with photopolymerizable liquid resins has been integrated into applications, predominantly in dentistry [30]. ABS-like SLA and ABS–FDM offer enhanced performance characteristics compared to other available counterparts. Traditionally manufactured plastics, such as polystyrene or polypropylene, may lack the mechanical strength and precision required for high-demand applications in dentistry.

Three-dimensional printing of dental arch models has revolutionized dental practice, providing an innovative approach to creating accurate and personalized solutions [31]. Using 3DP technology, medical professionals can reproduce intricate details of the patient’s dental arch, facilitating and accelerating the planning of treatments such as implants, prosthetics, and orthodontics [31,32]. Additionally, 3DP allows for the quick and efficient production of personalized models, reducing patient wait times and improving the dental office experience. Advanced three-dimensional printing techniques not only elevate the quality of dental care but also represent a significant step towards personalization and precision in clinical practice, promoting more effective and satisfactory results [33,34].

This study investigates the differences in mechanical properties between ABS samples produced using ABS filaments via FDM and ABS-like resin via stereolithography (SLA). The overarching objective is determining the process–property interrelationship of ABS and ABS-like resin to deliver superior performance for specific applications, particularly dental treatments. The significance of this study lies in its potential to provide valuable insights into the comparative performance of FDM and SLA techniques within the dental field. As 3D printing becomes increasingly crucial in dentistry, understanding the impact of each method on material properties can help optimize the selection of techniques and materials, thereby enhancing the precision and effectiveness of dental solutions. This study aims to thoroughly analyze these polymers and identify the more suitable method for dental applications, contributing to technological advancements and clinical practice improvements.

## 2. Materials and Experimental Methods

### 2.1. Materials and Processing

**Figure 1:**
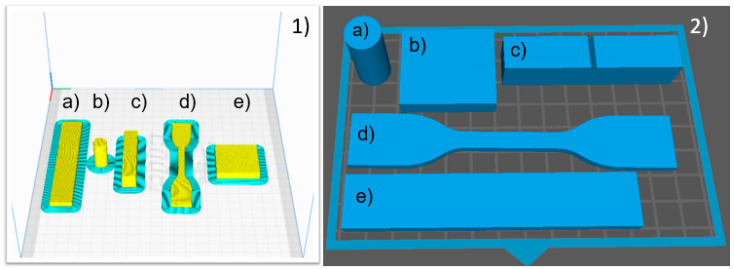
(**1**) FFF-slicing process with Ultimaker Cura software (version 4.2). (**a**) 3-point flexural resistance, (**b**) compression, (**c**) izod impact, (**d**) tensile, (**e**) shore hardness samples. (**2**) The respective SLA-slicing process with Chitubox.

The ABS filaments for the FFF technique were acquired from 3D Fila (Belo Horizonte, Brazil), with a diameter of 1.75 mm and a density of 1.06 g/cm^3^. The samples were manufactured using the filament printer Creality 3D Ender 3 V2 (Shenzhen Creality 3D Technology Co., Shenzhen, China). The sample geometries were modeled in CAD software (Meshmixer version 3.5), then imported into the Cura slicer software (version 4.2) (Ultimaker, Utrecht, The Netherlands) to configure the parts for printing (Figure 1(1)). The printing parameters include layer height 0.16 mm, fill density 100%, zigzag fill pattern, printing temperature 250 °C, print bed temperature 100 °C, print speed 80 mm/s, and nozzle diameter 0.4 mm.

The photocurable resin for the SLA technique came from the Elegoo (Shenzhen, China) and was processed by the Anycubic printer Photon Mono 4K (Anycubic, Shenzhen, China). This resin comprises polyurethane acrylate, acrylate monomers, and photo-initiators, which are marketed as comparable to the properties of ABS filaments used in FFF printing [20]. Recently, Singh et al. affirmed the similarities between FFF-printed ABS and SLA-fabricated ABS-like photocurable resin, reporting the dependence of the viscoelastic properties of these materials on their molecular structures [24]. The sample geometries were prepared for printing using a Chitubox slicing software (version 2.1) (Chitubox Inc., Shenzhen, China), Figure 1(2). The printing parameters included layer height 0.05 mm, normal exposure 4 s, and base layer exposure time 50 s. After printing, all SAL-based samples were rinsed with isopropyl alcohol and cured for 10 min under ultraviolet light in a wash and cure machine. The test procedures for the materials are presented in the following subsections.

### 2.2. Material Characterizations

The mechanical, durability, physical, and thermal properties of the 3DP materials herein were determined using several testing procedures. Table 1 summarizes the methods, parameters, standards, and equipment used in this research study to reveal the process-property interrelationship of 3DP ABS filaments and ABS-like resins.

#### Poisson’s Coefficient

**Figure 2:**
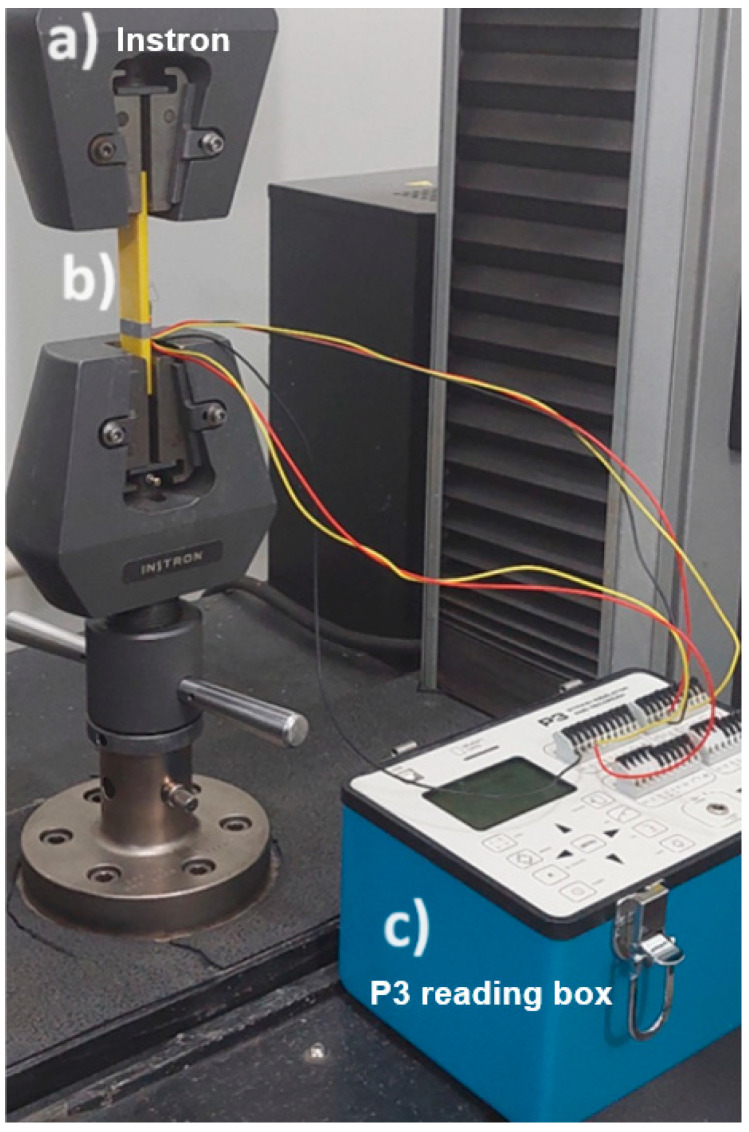
Poisson’s ratio test. (**a**) Instron load frame, (**b**) tested sample with strain grid, and (**c**) P3 strain gauge reading box.

The tensile test was carried out using the INSTRON-5582 universal testing machine, which attached clip-gage extensometers to the sample. The strain data were recorded using the P3 Strain Indicator and Recorder reading box (Figure 2). The samples were submitted to 300 N at a 0.5 mm/min loading rate following ASTM D638.

### 2.3. Statistical Analysis

After the data acquisition using the methods summarized in Table 2, the data were archived in a database and analyzed using the PAST program (version 4.12b). The data sets were compared using an analysis of variance (ANOVA) at a 5% significance level.

## 3. Results and Discussion

### 3.1. Mechanical Tests

At the onset, it is imperative to note that the SLA-fabricated specimens were 100% filled, similar to the infill percentage chosen in FFF printing, to normalize the printing parameters since internal infill affects the mechanical response of the polymers. The hardness test results were Shore 75D for the samples manufactured using FFF and SLA (Table 2), irrespective of the manufacturing method and composition. The results indicate the printed samples are insensitive to the manufacturing process (e.g., thermal treatment vs. ultraviolet curing) and molecular composition (ABS filaments and ABS-like photocurable resin) since the hardness test probes plasticity.

The Shore D hardness test was applied to samples after accelerated premature aging using UV light. The aging process significantly impacted the material processed by the SLA method, increasing Shore D hardness. The exposure to UV light alters the molecular structure due to the presence of the photo-initiator in the previously cured samples. The UV–polymer interactions increase the mechanical properties of the superficial layers on the sample, which is reflected in the increased Shore D hardness. Conversely, the material processed by the FFF method showed no significant changes in Shore D hardness, density, or weight after UV aging, indicating enhanced stability under accelerated aging conditions.

**Figure 3:**
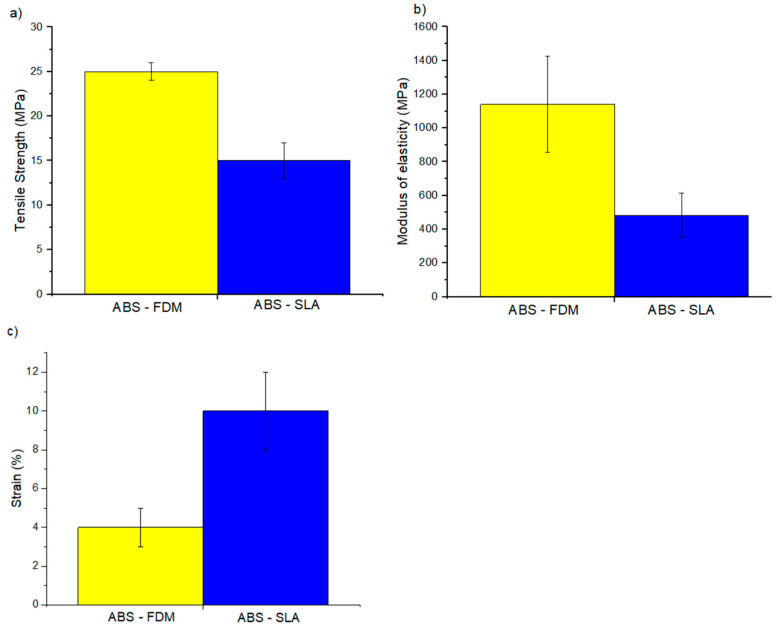
Tensile properties: (**a**) tensile strength, (**b**) modulus of elasticity, and (**c**) strain.

**Figure 4:**
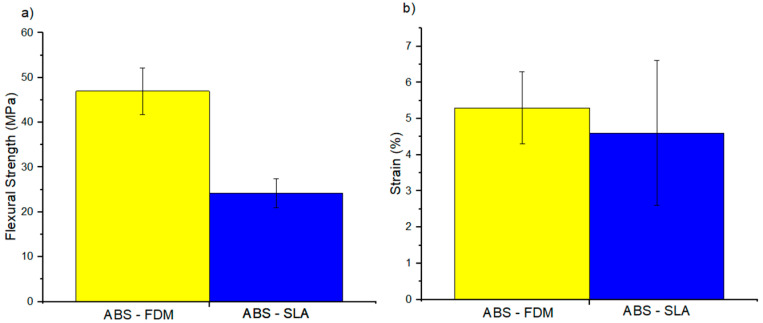
Flexural properties: (**a**) flexural strength and (**b**) strain.

**Figure 5:**
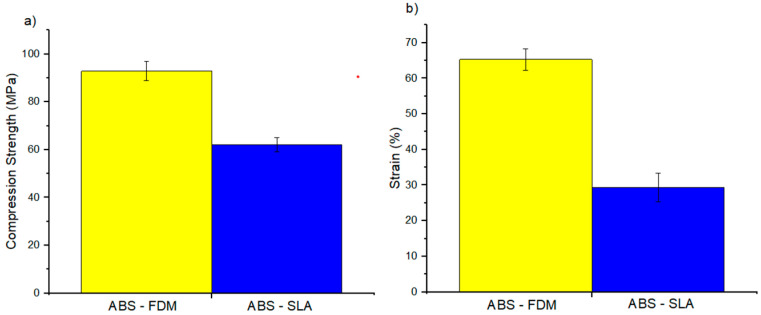
Compression properties: (**a**) strength and (**b**) strain.

Table 3 shows the tensile, flexural, and compressive properties and the *p*-value obtained from the ANOVA analysis. Samples produced by FFF were significantly stiffer and stronger, while those produced by SLA were ductile (Figure 3, Figure 4 and Figure 5). The dichotomy in the results is due to variations in chemical composition with additives such as acrylate monomer, which controls the polymer network during resin fabrication. This adjustment capability allows for optimizing the mechanical properties of resins produced by SLA, resulting in more ductile materials. These findings are consistent with previous studies cited in the literature [46,47]. After compression testing, samples produced by SLA exhibited fragmentation, indicating a more brittle behavior. In contrast, samples produced by FFF showed fractures due to flattening, suggesting a different stress distribution and a more robust mechanical response.

Tensile strength in plastics is influenced by various factors beyond just chemical composition [48,49]. While changes in chemical composition, such as adding reinforcing agents or modifying polymer chains, can enhance tensile strength, other elements also play a critical role. Introducing fillers, such as ceramics or fibers, can significantly increase tensile strength by reinforcing the polymer matrix and improving its structural integrity [50,51]. These fillers help distribute stress evenly across the material, reducing the likelihood of failure. Additionally, the degree of crystallinity and molecular alignment within the polymer can affect tensile strength. Materials with higher crystallinity or well-aligned polymer chains generally exhibit better tensile properties due to more effective load transfer within the material [52].

The modulus of elasticity reported herein is lower than that reported by the manufacturer while being consistent with the results of Szykiedans and Credo [53] comparing the tensile properties of FFF and SLA samples.

**Figure 6:**
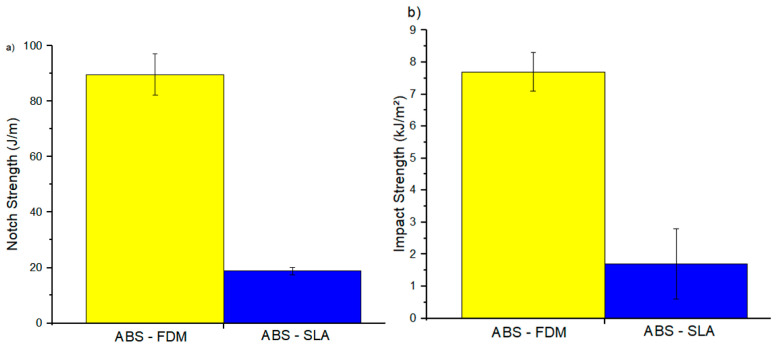
Izod impact resistance properties: (**a**) notch and (**b**) impact strength.

**Figure 7:**
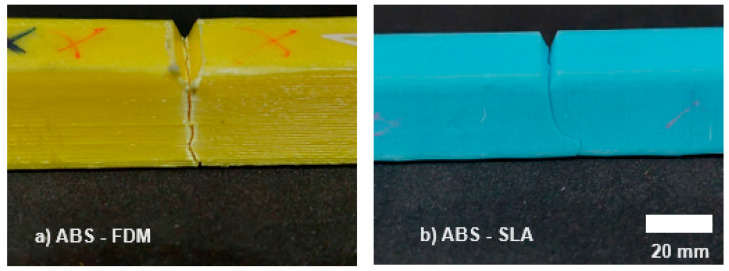
The fracture behavior pattern of specimens in the Izod test, (**a**) ABS–FFF, and (**b**) ABS–SLA.

Izod impact energies with their respective standard deviations are listed in Table 4. Based on the results of the IZOD test (Figure 6), the material behavior followed that observed in the tensile test (Table 2). The FFF samples have more significant impact resistance than the SLA counterparts. Figure 7 shows the fracture behavior as a function of the printing method and material composition. The data in this work corroborates those presented in the literature as a function of the 3D printing process, in which materials processed via FFF have better mechanical properties than their SLA counterparts [46,47,53]. For example, Górski et al. [54] and Shilpa et al. [55] ascertained the inferior mechanical resistance of samples made using SLA-printable, photocurable resin.

ABS–FDM exhibits superior mechanical properties compared to ABS-like SLA due to the intrinsic characteristics of the materials (e.g., molecular structure and composition) as a function of respective processing methods. ABS, a thermoplastic used in FFF, ensures strong interlayer bonding as the material melts and fuses during printing, enhancing tensile strength and impact resistance. In contrast, the ABS-like resin used in SLA, a thermoset material, cures into a rigid, cross-linked network that is more brittle and less impact-resistant. The FFF process benefits from continuous filament layers that bond effectively, whereas the SLA process results in a more brittle structure due to its curing mechanism.

The results of the Shore D hardness test showed similar microscale hardness, irrespective of the 3D printing type. The chemical interactions between particles, threads, and main compositions in plastics play a crucial role in determining their properties. In thermoplastics ABS used in FFF, long polymer chains interact and entangle, allowing enhanced bonding between layers and greater mechanical strength. Conversely, in thermoset resins used in SLA, chemical cross-linking occurs during curing, creating a rigid network that enhances hardness but introduces brittleness. Additives and fillers also influence the properties of both plastics by modifying the polymer network, imparting characteristics such as flexibility and strength.

**Figure 8:**
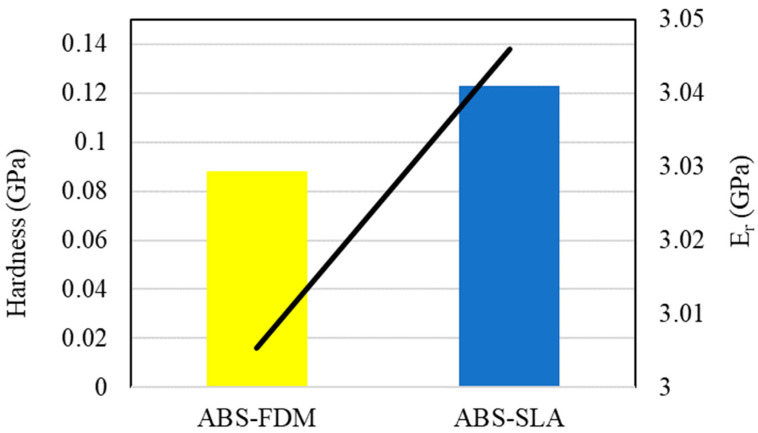
Nanoindentation results for ABS–FDM and ABS–SLA samples.

However, the nanoindentation test with a smaller indenter tip improves the accuracy of the results (Figure 8). The FFF sample suffers from layer adhesion and thermal history during printing. It is more prone to voids between the extrusion lines, as demonstrated in the Confocal Laser Scanning Microscope test, discussed next. The presence of manufacturing imperfections can affect its hardness. In contrast, the SLA sample is produced with improved layer adhesion given the molecular and mechanical interlocking during the photocurable printing process (Table 3), resulting in higher hardness. The SLA samples also exhibited a higher reduced modulus, as shown in Figure 8, corroborating the results in Table 2 from the tensile test.

The processing parameters used to produce plastics substantially impact their mechanical properties [56]. For instance, in FDM, factors such as printing temperature, layer height, and extrusion speed influence the bonding between layers and the overall mechanical performance of the printed object. Higher printing temperatures can enhance layer adhesion and reduce internal voids, improving tensile strength and impact resistance [57,58,59]. In contrast, in stereolithography (SLA), parameters like curing time, light intensity, and resin composition affect the polymerization process, leading to hardness and brittleness variations. Precise control over these parameters ensures optimal material properties by affecting the degree of cross-linking and structural integrity of the final product [60,61,62]. Thus, fine-tuning the process parameters is essential for achieving desired mechanical properties in thermoplastic and thermoset materials.

### 3.2. Characterization of the Microstructure and Thermal Attributes

Through the analysis of the Confocal Laser Scanning Microscope, it was possible to verify the layer height of 34.3 μm based on one voxel, confirming the precision quality of the resin samples. For filament samples with 100% internal filling, an analysis showed 390.6 μm for the layer height, which also can be visualized by the scanning electron microscope (SEM) analysis.

**Figure 9:**
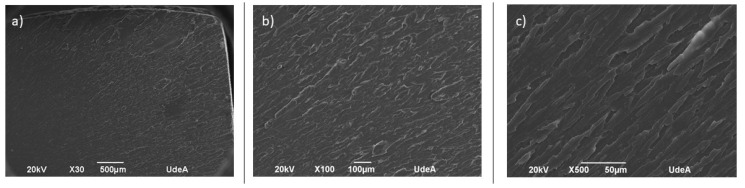
SEM micrographs of fractured surface for ABS–SLA samples at magnifications of (**a**) 30×, (**b**) 100×, and (**c**) 500×.

**Figure 10:**
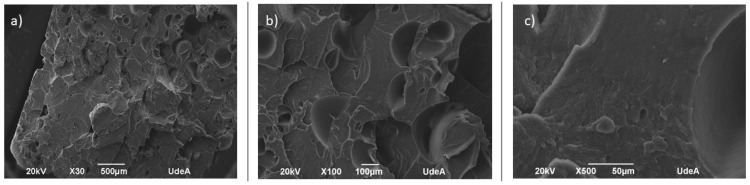
SEM micrographs of fractured surface for ABS–FFF at magnifications of (**a**) 30×, (**b**) 100×, and (**c**) 500×.

In the SEM analysis, the samples evince cracks due to flexural loading, which initiates and becomes arrested within the continuum, creating fragmentation. In SLA samples (Figure 9), small cracks and voids were observed, resulting in flat fracture surfaces, suggesting that the material had no resistance against the applied load. In FFF samples (Figure 10), the SEM micrographs show the presence of pores and the roundness of different roads lain during the 3D printing process.

**Figure 11:**
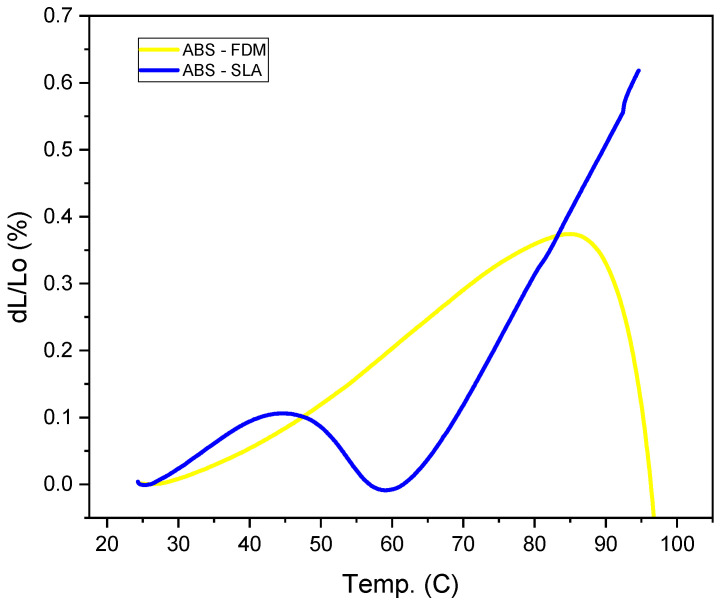
Dilatometry results for the sample made by ABS–FFF and ABS–SLA.

Thermal expansion values were calculated up to 100 °C and reported in Figure 11. The curves showed similar dilatometric behaviors, with slight accentuations of the phenomena for the SLA sample (Figure 11) and slight contraction at 47 °C. The FFF samples experienced an intense contraction at 85 °C, where the shrinkage can lead to delamination between the layers, leading to printing failure. For FFF 3D printing, the linear expansion rate of the materials is a critical factor in affecting the dimension of the products.

**Figure 12:**
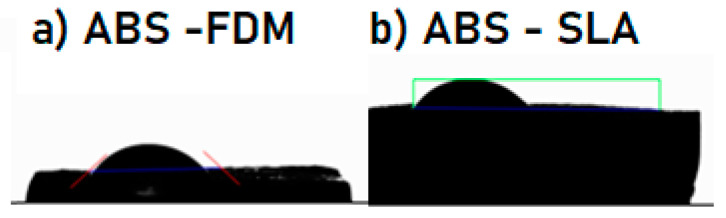
KSV K100 drop tensiometer for contact angle assessment, (**a**) ABS–SLA and (**b**) ABS–FFF.

To perform the wettability test using the KSV K100 drop tensiometer (KSV Instruments, Espoo, Finland), a clean and dry substrate was used, on which distilled water drops were applied. After dispensing the drops, the contact angles formed between the drops and the substrate were recorded using the manufacturer’s specific automatic software (Surfaceware version 9.9). The wettability test results show that ABS–SLA and ABS–FFF are hydrophilic materials. The average contact angle (Figure 12 and Table 5 for ABS–SLA is 44.58° and 35.25° for ABS–FFF samples, indicating good wettability that facilitates the adhesion of liquids to the surface. Hydrophilicity can influence interlayer adhesion, resulting in more stable and cohesive prints. Hydrophilic surfaces are easier to clean and sterilize, as water and other cleaning liquids spread more quickly.

**Figure 13:**
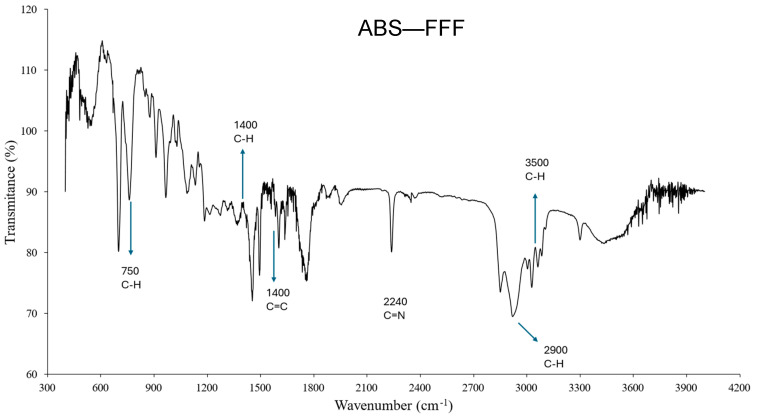
FTIR spectrum of ABS–FFF.

**Figure 14:**
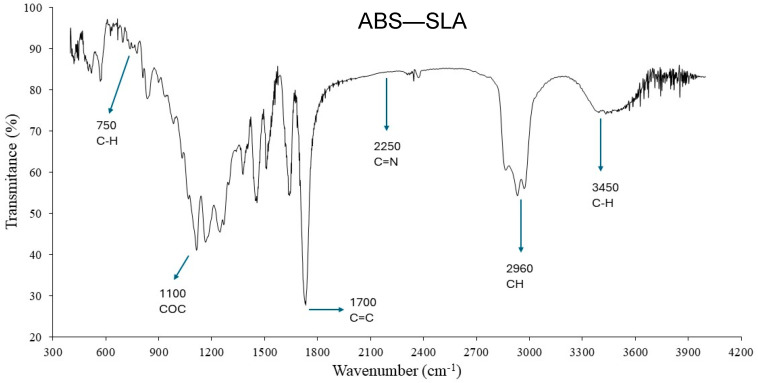
FTIR spectrum of ABS–SLA.

Figure 13 shows the FTIR (Fourier transform infrared spectroscopy) spectrum of the ABS-based samples, while Figure 14 reports the FTIR spectrum for ABS-like samples printed using the SLA process. The FTIR spectra reported herein confirm the distinct structure and molecular structure discussed in Section 2 above, where the filament is indeed the ABS terpolymer, and the photocurable resin is an acrylate-based polymer.

### 3.3. Application for Odontology

**Figure 15:**
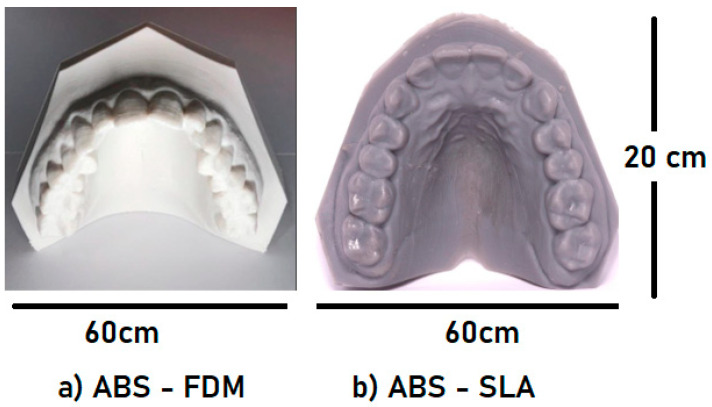
Dental arch models and surgical guides with (**a**) ABS–FFF and (**b**) ABS–SLA.

The union of knowledge between the areas of engineering and applied dentistry and recent research using computational tools has become increasingly relevant in the scientific world. The reliability of these findings, demonstrated by their applicability, versatility, and fidelity of results [63,64,65], instills confidence in their potential impact. With the data obtained and analyzed herein, it is possible to use printable ABS polymer in the dental field. The additive manufacturing methods (FFF and SLA) yielded desirable tensile strength and elastic modulus samples. Therefore, these processes are suitable for functional prototypes, such as dental arch study models (Figure 15a,b) and surgical guides, which require high resistance to tension with minimal deformation [65,66,67].

**Figure 16:**
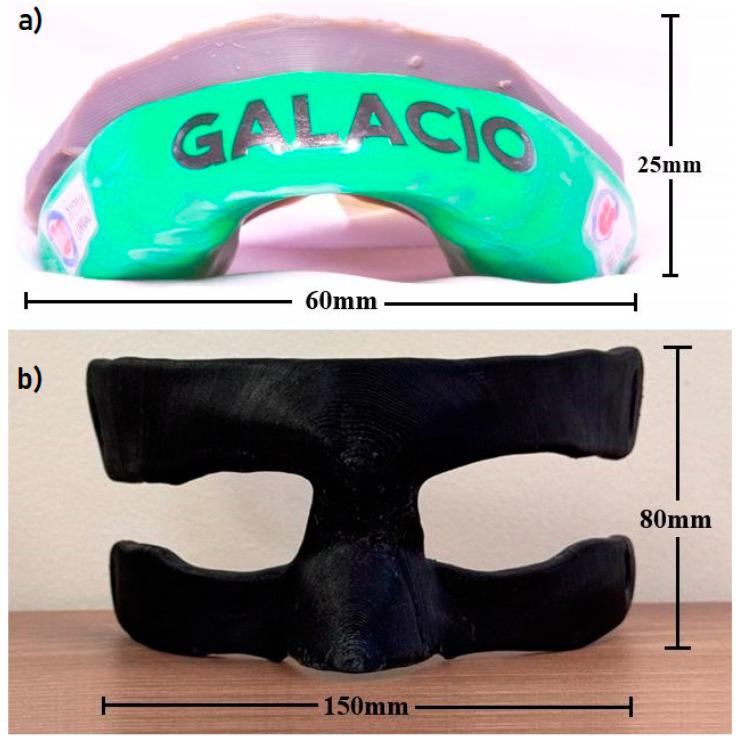
(**a**) Model ABS—SLA fittings/templates for the occlusal device, and (**b**) mouthguard facial protectors using ABS—FFF.

It is common in dentistry to require details of dental anatomy and the need for precision for fittings/templates for provisional prostheses, occlusal plates (Figure 16a), and dental prostheses. The microstructural characterization shows that the SLA processing method has better voxel precision [65,66,67]; however, if a model is necessary for just studying the dental arch or initial documentation of orthodontic treatment, FFF 3D printing is recommended.

Three-dimensional-printed facial protectors using the FFF method are notable since high-performance athletes require advanced face protective devices to accelerate injury recovery. When athletes suffer a fracture in their facial bones, they are forced to stay away from their activities until they recover in around 30 days [68,69]. However, fabricating patient-specific facial protectors results in immediate reintegration into training, allowing a faster return to the athlete’s physical condition [69,70]. Fowell and Earl [71] emphasized that facial protectors protect the athlete’s face from an orofacial injury that can prevent the bone from refracture or displacement. Hence, 3D-printed ABS can be a competitive material candidate for density applications, potentially replacing other polymers. For example, studies evaluating ethylene vinyl acetate copolymer (EVA) in mouth and face protectors using finite element analysis and mechanical tests prove the effectiveness of the protection [71,72,73,74,75,76]. EVA-protective guards must be the same as those used to make mouth guards, which meet specific standards for contact with the skin for a prolonged period. This material’s monomer composition varies, determining different properties such as flexibility according to the brand sold. Several commercialized EVAs allow for prolonged contact with the skin and mucous membranes. Nonetheless, ABS is a promising material for this application (Figure 16b), and the data highlighted in the study show that the material has the same tensile strength and a similar modulus of elasticity. Future studies are still necessary with the data obtained in the survey for simulations using the finite-element method for impact assessment to validate the application and the evaluation of irritability from prolonged contact with the mucosa and skin.

## 4. Conclusions

This study revealed that, although the Shore D hardness is similar for ABS manufactured using fused filament fabrication (FFF) and stereolithography (SLA) methods, the FFF process provides significantly superior mechanical properties compared to SLA, while SLA processing excels in precision. The data analysis indicates that the ABS filament used in FFF 3D printing is a thermoplastic polymer, whereas the ABS-like photocurable resin is a thermoset, predominantly polyurethane acrylate. Based on the obtained data, it is concluded that printable ABS is suitable for dental applications, such as models and surgical guides, using both SLA and FFF methods. Furthermore, FFF can be used for sports face shields and models with lower precision requirements. Future studies should validate these applications through simulations using finite-element methods for impact assessment and analysis of material irritation from prolonged contact with mucosa and skin.

## Figures and Tables

**Table 1:** Characterization of materials, methods, and standards (e.g., American Society for Testing and Materials, ASTM).

Test	Standard	Machine	N° of Specimens	Standard
Shore D Hardness and Accelerated Aging	ASTM D2240 [35]. ASTM G-53 [36]	Shore D Hardness tester UV Accelerated Machine	7	Five indentations in an “X” format were performed, with a minimum distance of 12 mm from the edges and 6 mm between the test points. A representative sample of each material was subjected to a UV aging process simulating a cycle of 120 h.
Tensile Test	ASTM D638 [37]	Universal Tensile Tester, Instron 5582	7	With a machine speed of 0.5 mm/min [38]
Impact Test Izod	ASTM D256-10 [39]	PANTEC Pendulum XC-50	7	Using a 22 J hammer
Flexural resistance 3 points	ASTM D790 [40]	Universal Tensile Tester, Instron 5582	7	3-point bending using a descent speed of 0.5 mm/min, and a distance of 80 mm.
Compression Test	ASTM D695 [41]	Universal Tensile Tester, Instron 5582	7	Cylinders with dimensions of 12.7 mm and a length of 25.4 mm
Poisson coefficient	ASTM E132-86 [42]	Universal Tensile Tester, Instron 5582	7	Session 2.2.1
Characterization of the microstructure	-	JEOL JSM 6700R	1	Scanning electron microscopy (SEM)
Density	ASTM E694-15 [43]	Mettler Toledo balance	7	Archimedes principle
Confocal Microscopy	-	OLYMPUS LEXT OLS4000	1	Addition to a magnification amplitude of up to 17,091×
Dilatometry	ASTME228 [44]	Dilatometer Netzsch DIL 402 PC	1	5 °C to 100 °C, with a heating rate of 2 °C/min
Wettability	ASTM D7334-08 [45]	Drop tensiometer KSV K100	1	Clean and dry substrate on which drops of distilled water were applied with a temperature control of 20°
FTIR	-	Shimadzu IR-Affinity-1 spectrophotometer	1	Range 4000–400 cm^−1^, resolution 4 cm^−1^, and 32 scans.
Nanoindentation	-	MicroMaterials NanoTest Vantage	1	Ten randomly selected indentation sites at 100 mN

**Table 2:** Hardness test results.

	FFF	SLA
Shore Hardness (Shore D)	75D ± 0.5	75D ± 0.2
Weight Pre-Aging (g)	11.87 ± 0.45	15.89 ± 0.76
Density Pre-Aging (g/cm^3^)	1.14 ± 0.11	1.2 ± 0.22
Shore Hardness Accelerated Post Aging (Shore D)	76.5D ± 0.3	80D ± 0.5
Weight After Early Aging (g)	11.48 ± 0.89	14.89 ± 0.76
Post Premature Aging Density (g/cm^3^)	1.13 ± 0.09	0.9 ± 0.13

**Table 3:** Properties obtained in mechanical characterization from the tensile, flexion, and compression tests.

Parameter	ABS—FFF	ABS—SLA	*p*-Value
Tensile strength	25 ± 0.9 MPa	15 ± 2.4 MPa	<0.001
Tensile modulus	1140 ± 285 MPa	482 ± 131 MPa	<0.001
Tensile strain	4 ± 1%	10 ± 5%	0.016
Flexural strength	46.96 ± 5.2 MPa	24.2 ± 3.2 MPa	<0.001
Flexural strain	5.28% ± 1%	4.26% ± 2%	0.020
Compressive strength	92.9 MPa	62 MPa	<0.001
Compression strain	65.28% ± 3%	29.35% ± 4%	0.014
Poisson’s ratio	0.38	0.41	------

**Table 4:** IZOD test results.

	ABS—FFF	ABS—SLA	*p*-Value
Notch Strength	89.7 ± 7.5 J/m	18.8 ± 1.36 J/m	<0.01
Impact Strength	7.7 ± 0.6 kJ/m^2^	1.7 ± 1.1 kJ/m^2^	<0.01

**Table 5:** Wettability test results.

Sample	Average Contact Angle	Classification
ABS–FDM	35.25°	Hydrophilic
ABS–SLA	44.58°	Hydrophilic

## Data Availability

The original contributions presented in the study are included in the article, further inquiries can be directed to the corresponding author.
